# Association between habitual use of coping strategies and posttraumatic stress symptoms in a non-clinical sample of college students: A Bayesian approach

**DOI:** 10.1371/journal.pone.0228661

**Published:** 2020-02-06

**Authors:** Arthur Viana Machado, Eliane Volchan, Ivan Figueira, Carolina Aguiar, Mariana Xavier, Gabriela G. L. Souza, Ana Paula Sobral, Leticia de Oliveira, Izabela Mocaiber

**Affiliations:** 1 Laboratory of Behavioral Neurophysiology, Physiology and Pharmacology Department, Biomedical Institute, Federal Fluminense University, Niterói, RJ, Brazil; 2 Laboratory of Cognitive Psychophysiology, Department of Natural Sciences, Institute of Humanities and Health, Federal Fluminense University, Rio das Ostras, RJ, Brazil; 3 Laboratory of Neurobiology II, Biophysics Institute, Federal University of Rio de Janeiro, RJ, Brazil; 4 Institute of Psychiatry, Federal University of Rio de Janeiro, Rio de Janeiro, RJ, Brazil; 5 Laboratory of Psychophysiology, Department of Biological Sciences, Federal University of Ouro Preto, Ouro Preto, MG, Brazil; 6 Department of Engineering, Institute of Science and Technology, Federal Fluminense University, Rio das Ostras, RJ, Brazil; RWTH Aachen, GERMANY

## Abstract

The present study investigated the influences of coping styles on posttraumatic stress symptoms (PTSS) among a sample of non-clinical college students who were exposed to traumatic events. Ninety-nine college students participated in the study. However, the sample used in the analyses consisted of only 37 participants who fulfilled the DSM-IV criterion A for Posttraumatic Stress Disorder (PTSD) diagnosis. The PTSD Checklist–Civilian Version (PCL-C) and the Brief COPE were used to assess the participants’ PTSS and habitual use of coping strategies, respectively. Bayesian and frequentist correlations showed that emotion-focused coping style was negatively associated with PTSS, while dysfunctional coping style was positively related to PTSS. In the subsequent linear regression on both statistical framework, dysfunctional coping was the only consistent variable predicting more PTSD symptoms. The findings presented here show that lower use of adaptive coping (emotion-focused) and higher use of dysfunctional coping styles on a daily basis are associated to PTSS severity in a non-clinical sample of college students. According to the Bayesian approach, which permits more generalization of data, dysfunctional coping style is determinant to higher levels of PTSS. These findings add new data to the body of research that highlight the critical role of distinct coping strategies in the severity of PTSS.

## Introduction

A typical response to a threatening event involves a constellation of behavioral and physiological reactions and, in the aftermath, when danger has finished, a recovery to basal levels [1]. Exacerbated reactivity and failure to recover after a traumatic event are associated with posttraumatic stress disorder (PTSD) occurrence [2,3,4].

PTSD is characterized by symptoms that are developed at least one month after an event which involves death, serious injury or sexual violence (A criterion). The individual must experience, witness or learn about traumatic events of an emotional close person, or must be repeatedly exposed to aversive details of a traumatic event, and the symptoms must cause functional impairment. There’s a myriad of symptom in PTSD such as intrusions related to the traumatic event, avoidance associated with internal or external trauma-related stimuli, negative changes in cognition and mood, and changes in arousal and reactivity [5,6].

Considering the literature, emotion dysregulation and the use of poor and inadequate coping styles while facing a stressor represent risk factors that enhance the probability of developing posttraumatic stress symptoms (PTSS) [7–20]. According to Lazarus and Folkman [21], coping interferes with one’s ability to resist to a trauma, probably attenuating stress responses. In fact, the use of adaptive coping skills is part of the treatment of patients with PTSD and helps to alleviate PTSS following a traumatic experience [22]. Coping refers to cognitive and behavioral efforts to manage the internal and external demands of the interaction between the individual and the environment [21]. Basically, coping styles can be classified into problem-focused coping (e.g., dealing with stress sources and taking proactive steps to change them) and emotion-focused coping (e.g., regulating one’s emotion to reduce stress) [23–25]. Additionally, coping styles can also be classified as adaptive/functional and maladaptive/dysfunctional [26]. Adaptive coping styles (e.g., cognitively reframing a stressor) are strategies focused on reducing stress or eliminating the stressor and are related to positive outcomes such as optimism, high self-esteem, and resilience. In contrast, dysfunctional coping styles (e.g., denial) have questionable value in reducing stress or eliminating the stressor, and they are related to poor outcomes such as high trait anxiety, low self-esteem, low optimism, low resilience, and high PTSS severity [9,16,26].

As previously mentioned, some studies have reported an association between coping styles and PTSS. For example, Cofini et al. (2015) showed that adults with PTSS have used more maladaptive coping strategies (denial, venting, behavioral disengagement, and self-blame) in dealing with an earthquake’s stressful impact [9]. Other studies reported a strong association between avoidant coping styles and higher levels of PTSS in female victims of physical and sexual assault [13,15,18]. In the same vein, studies showed that individuals with emotion regulation difficulties scored higher on PTSS [12,14,17].

Several studies have reported the relationship between coping styles and PTSS in clinical populations with PTSD [27–29], but data are scarcer regarding emotional dysregulation and mechanisms of coping in non-clinical samples. To our knowledge, only Schnider et al. (2007) and Dworkin et al. (2018) investigated the association between habitual coping styles and PTSS severity in non-clinical college students, showing that avoidant emotion coping and lack of social support predicted PTSS, respectively [16,30]. These non-clinical samples may be advantageous since it minimizes confounding factors such as comorbidities and medication use, allowing a more precise and accurate comparison with data yielded in different studies.

It is important to note that the number and severity of mental disorders among the young population, specifically college students, seem to be increasing [31]. The most common mental health problems among college students are anxiety, mood disorders and substance abuse [32]. According to the National Institute of Mental Health, having a history of mental illness or substance abuse is a risk factor for the development of PTSD. Moreover, most lifetime mental disorders have their first onset at the age of 24 years [31,33], so choosing appropriate ways to cope is an important tool to attenuate the impact of daily routine stressors that may contribute to the development of PTSS, especially in regard to college students who often are young. Several studies have shown that reappraisal, considered an adaptive coping strategy, can downregulate brain responses to negative stimuli [34–35]; this is especially important in those with higher PTSD symptom severity since they often show greater electrocortical and brain hyperactivation in response to negative traumatic and non-traumatic stimuli [36–37]. Thus, our main goal was to investigate whether the habitual use of different coping styles would be associated with PTSS in a non-clinical sample. Our hypothesis was that habitual use of adaptive coping would be associated with better outcomes, while dysfunctional coping would be related to higher PTSS. Our secondary goal was to compare two statistical approaches: the Frequentist and Bayesian analyses. This is important because one current issue in our scientific community is the replication of findings due to low power and the use of statistical methods that are not adequate for the analyses [38–39].

## Material and methods

### Participants

A total of 99 undergraduate students participated in this study (57 women, mean age = 23.06, SD = 3.35). However, the sample used in the analyses included 37 students (20 women, mean age = 22.86, SD = 2.90) whose trauma met criterion A of DSM-IV, which can be defined as “experience, witness or learn about death or threatened death, actual or threatened serious injury, or actual or threatened sexual violence”. Although these 37 students had a trauma that met criterion A of DSM-IV none of them had a diagnosis of PTSD confirmed by a psychiatry. Our sample was composed of Nursing students (n = 22); Psychology students (n = 7); Computer Science students (n = 3); Engineering students (n = 2); and Arts and Cultural Studies students (n = 1); 5% (n = 2) did not report their courses. The participants were recruited on campus and during classes, those who were interested in participating in the study provided their names and cellphone contacts. Exclusion criteria were: being under any pharmacological treatment (except contraceptives) and report having any diagnosis of psychiatric, cardiac, or psychological disease. None of the participants reported being a smoker.

### Psychometric questionnaires

Posttraumatic Stress Disorder Checklist–Civilian Version (PCL-C) [40–41]: The PCL-C is an instrument used to evaluate PTSD symptoms. In addition to its use for tracking possible cases of PTSD, the scale can also be used to evaluate the severity of symptoms. Before answering the PCL-C, the participants had to describe the index trauma to which the symptoms were rated. The index trauma was the worst traumatic event to which the participant had been exposed. The question was “what was the most severe traumatic event in your life?” Hence, they completed the PCL-C with this potential traumatic event in mind. They also had to estimate when they were exposed to this index trauma. The PCL-C is a Likert scale (1 to 5 points) with 17 items distributed in three groups of PTSD symptoms (criteria B, C and D) from the DSM-IV [40]. The PTSD symptoms consist of persistent reexperience of traumatic event (criterion B), persistent avoidance of stimuli associated with the trauma and numbing of general responsiveness (criterion C), and persistent symptoms of increased arousal (criterion D) [6]. In addition to the groups of symptoms, the PCL-C has a cut-off score of 50 points for tracking possible diagnosis, which means that individuals scoring more than 50 points may have PTSD [42]. However, Blanchard et al. (1996) reported that a cut-off score of 44 points was associated with increased specificity and sensitivity [42], which improved diagnostic efficiency.

Brief COPE [43–44]: The Brief COPE was created by Carver (1997) to assess a variety of coping styles. The scale consists of 28 questions distributed into 14 subscales in a Likert-scale format (0 to 4 points). Each subscale contains two questions and represents a conceptually different coping style that can be functional or dysfunctional. As in other studies [11,45–46], we divided the whole scale into three clusters: (a) problem-focused coping: active coping, planning, and instrumental support; (b) emotion-focused coping: emotional support, religion, positive reframing, acceptance, and humor; and (c) dysfunctional coping: self-blame, venting, denial, self-distraction, behavioral disengagement, and substance use. The content of the 14 items of the Brief COPE are as follows: (1) active coping: “concentrating my efforts on doing something about the situation I’m in” / “taking action to try to make the situation better”; (2) instrumental support: “trying to get advice or help from other people about what to do” / “getting help and advice from other people”; (3) planning: “trying to come up with a strategy about what to do” / “thinking hard about what steps to take”; (4) acceptance: “accepting the reality of the fact that it has happened” / “learning to live with it”; (5) emotional support: “getting emotional support from others” / “getting comfort and understanding from someone”; (6) humor: “making jokes about it” / “making fun of the situation”; (7) positive reframing: “trying to see it in a different light to make it seem more positive” / “looking for something good in what is happening”; (8) religion: “trying to find comfort in my religion or spiritual beliefs” / “praying or meditating”; (9) behavioral disengagement: “giving up trying to deal with it” / “giving up the attempt to cope”; (10) denial: “saying to myself this isn’t real” / “refusing to believe that it has happened”; (11) self-distraction: “turning to work or other activities to take my mind off things” / “doing something to think about it less, such as going to movies, watching TV, reading, daydreaming, sleeping, or shopping”; (12) self-blame: “criticizing myself” / “blaming myself for things that happened”; (13) substance use: “using alcohol or other drugs to make myself feel better” / “using alcohol or other drugs to help me get through it”; and (14) venting: “saying things to let my unpleasant feelings escape” / “expressing my negative feelings” [43]. One important thing to note is that in our study, we instructed the subjects to answer the Brief COPE while thinking about events that occur on daily basis and not a specific event. Thus, the score refers to habitual coping strategies.

### Procedures

Each experiment was performed individually in a specially prepared room in the laboratory of Cognitive Psychophysiology. Initially, arriving at the laboratory, the participants read and filled the consent form and a personal form containing information about medical conditions, pharmacological treatments, etc. After filling out these forms, the participant was oriented to sit in front of a computer screen to answer the following psychometric questionnaires on Google forms: Brief COPE and PCL-C (Posttraumatic Stress Disorder Checklist–Civilian Version). This study was conducted with the formal approval of the Ethics Committee of the School of Medicine/Antônio Pedro University Hospital, Fluminense Federal University.

### Statistical analysis

Descriptive analyses of sample characteristics and psychometric scales were performed. Bivariate correlation (Pearson) analyses were conducted to measure the relationship between the three clusters of coping strategies and PTSD symptoms. We also carried out correlation analyses between PTSD symptoms criteria and coping strategies. Moreover, correlation tests between PTSS and each Brief COPE subscale were performed. A linear regression model was fitted to investigate the association between Brief COPE (independent variable) and PCL-C (dependent variable) scores. For all frequentist analyses we considered a p-value of less than 0.05. In addition to the classical frequentist approach, we used a Bayesian approach of the correlation test [47] to investigate the association between coping strategies and PTSD symptoms, since it is less dependent on sample size and allows inference on both alternative and null hypotheses. This type of analysis gives us the ability to evaluate the posterior distribution of a parameter and better address sample size problems, since maximum likelihood estimation can be quite poor to take effect on small samples [48]. In addition, the Bayesian correlation test assumes a bivariate *t* distribution in the MCMC (Markov Chain Monte Carlo) sampling process and it is more robust to outliers [49], which is a common problem in small samples. Moreover, in addition to the Bayesian correlation test, we also performed a Bayesian approach of the linear regression to evaluate the posterior distribution of the beta coefficients and compare with the frequentist model. Both analyses were performed using a MCMC algorithm with 10.000 iterations. All Bayesian and frequentist analyses were conducted in the R statistical software [50] and JASP [51]. We used the following packages for the Bayesian analyses: *BayesianFirstAid*, *BayesFactor*, and *bayestestR* [47, 52–54]. For conventional and practical purposes, we used the HDI (Highest Density Interval) + ROPE (Region of Practical Equivalence) decision rule of 0.1 (-0.1, 0.1), which is small effect for correlation analyses [55].

## Results

### Descriptive analysis

In the sample that trauma met criterion A of the DSM-IV (n = 37), the mean score on the PCL-C was 41.05 (SD = 17.19), and 43.24% (n = 16) scored above 44 points. 70% (n = 26) were indirectly exposed to the trauma, which means that they witnessed or learned about the traumatic event. The most frequent events reported were “death of a close relative” (49%), followed by “illness of a close relative” (13%), “sexual violence” (11%), “violent crime” (11%), “motor vehicle accident” (11%), and “other accidents” (5%). Regarding the time since trauma, most of the sample (97%) reported a trauma that occurred at least more than one month from the day they participated in the experiment. However, the exact time of the index trauma occurrence was not assessed.

### Frequentist correlation analysis

The severity of PTSS (indexed by the PCL-C total score) positively correlated with the dysfunctional coping cluster (p <0.001). In addition, the severity of PTSS negatively correlated with emotion-focused coping (p = 0.03)–Table 1. The correlation analysis conducted between each PCL-C criteria and each cluster of coping, showed that dysfunctional coping directly correlated with all PCL-C criteria: criterion B (p <0.001), criterion C (p <0.001), and criterion D (p = <0.001)–Table 2. Additionally, we found an inverse correlation between emotion-focused coping styles and criterion B (p = 0.01) and criterion C (p = 0.04).

**Table 1:** Correlation analysis between coping style clusters and PCL-C scores.

	PCL-C	
	r	p-value
**Problem-focused Coping**	-0.26	0.13
**Emotion-focused Coping**	-0.36	0.03
**Dysfunctional Coping**	0.70	< 0.001

Note. PCL-C = Posttraumatic Stress Disorder–Civilian Version.

**Table 2:** Correlation analysis between coping style clusters and PCL-C symptom clusters.

	Criterion B		Criterion C		Criterion D	
	r	p-value	r	p-value	r	p-value
**Problem-focused Coping**	-0.25	0.14	-0.27	0.11	-0.19	0.27
**Emotion-focused Coping**	-0.40	0.01	-0.35	0.04	-0.26	0.12
**Dysfunctional Coping**	0.56	< 0.001	0.60	< 0.001	0.79	< 0.001

Note. PCL-C = Posttraumatic Stress Disorder–Civilian Version.

Taking a closer look at the types of coping styles, we focused on each of the 14 components of the Brief COPE scale to further understand its impact on PCL-C scores–Table 3. We found that strategies such as active coping (p = 0.02), positive reinterpretation (p = 0.04) and humor (p = 0.007) had an inverse association with PTSS severity. In contrast, dysfunctional coping strategies such as self-blame (p < 0.001), venting (p = 0.005) and denial (p < 0.001) were positively correlated with PTSS.

**Table 3:** Correlation analysis between coping styles and PCL-C.

	PCL-C	
	r	p-value
**Active Coping**	-0.40	0.02
**Planning**	-0.24	0.14
**Instrumental Support**	-0.002	0.99
**Emotional Support**	-0.02	0.91
**Religion**	-0.25	0.13
**Positive Reinterpretation**	-0.34	0.04
**Self-Blame**	0.64	< 0.001
**Acceptance**	-0.06	0.72
**Venting**	0.45	0.005
**Denial**	0.56	< 0.001
**Self-Distraction**	0.26	0.12
**Behavioral Disengagement**	0.26	0.12
**Substance Use**	0.31	0.06
**Humor**	-0.43	0.01

Note. PCL-C = Posttraumatic Stress Disorder–Civilian Version.

### Linear regression analysis

To further investigate the association between the different strategies of coping and PTSS, we built a regression model with PTSS as the dependent variable and the three clusters of coping as possible predictors, we also entered gender as a confound variable–Table 4. The model provided a good fit, accounting for 57.16% of the variation in posttraumatic stress symptoms (R^2^ = 0.5716, F = 10.67, p < 0.001). Only dysfunctional coping (p < 0.001) was a significant predictor of PTSS severity.

**Table 4:** Frequentist linear regression analysis with PCL-C score as the dependent variable.

	$\hat{\beta }$	SE	R^2^	F
**Gender_female**	3.73	4.08	0.5716	10.67
**Dysfunctional coping**	1.75**	0.31		
**Emotion-focused coping**	-0.49	0.33		
**Problem-focused coping**	-0.43	0.76		

* p < 0.05

** p < 0.01

Note. PCL-C = Posttraumatic Stress Disorder–Civilian Version. SE = Standard Error.

### Bayesian correlation analysis

**Fig 1:**
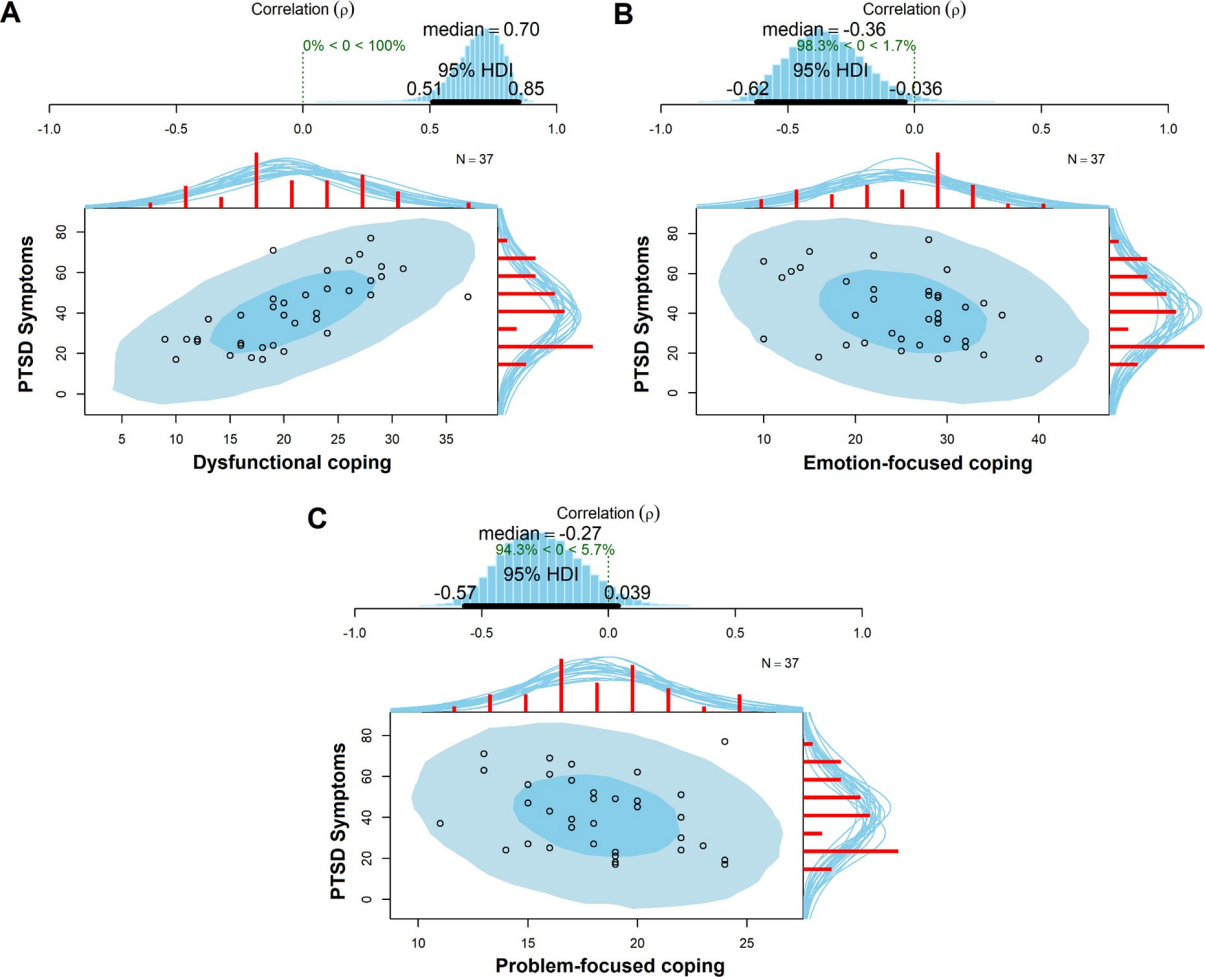
Posterior distributions of the correlation coefficients between PTSD symptoms and coping strategies clusters. Figures A, B and C displays a blue histogram on top showing the posterior distribution of the correlation (p) with a 95% highest density interval (HDI). On the bottom of each figure, we have the superimposed posterior predictive distributions, with the lighter blue showing the 95% highest density region and the dark blue showing the 50% highest density region. The red histograms on top x-axis and right y-axis show the marginal distribution of the data with a smatter of marginal densities drawn from the posterior.

The Bayesian correlation revealed an estimated correlation of the posterior distribution (p) of 0.71 between PTSD symptoms and dysfunctional coping style (95% CI[0.51, 0.85]), demonstrating that the correlation coefficient is more than 0 by a probability of 99.9% (Fig 1A). For the analysis between emotion-focused coping and PTSD symptoms, the Bayesian Pearson correlation test showed an estimated correlation of (p) –0.36 (95% CI [–0.63, –0.04]), suggesting that the correlation coefficient is less than 0 by a probability of 98.3% (Fig 1B). However, part of the 95% HDI falls into the Region of Practical Equivalence (ROPE), which means that it must be carefully evaluated. Although part of the 95% HDI falls into the ROPE, PTSD symptoms and problem-focused strategies had an estimated correlation (p) of –0.27 (95% CI[–0.55, 0.04]), suggesting that the correlation coefficient is less than 0 by a probability of 94.3% (Fig 1C).

The Bayesian approach was also applied to verify associations between each PCL criteria and coping style. It showed that dysfunctional coping style was positively associated with criterion B (estimated correlation (p) = 0.59, 95% CI[0.35, 0.79]), criterion C (estimated correlation (p) = 0.60, 95% CI[0.36, 0.79]), and criterion D (estimated correlation (p) = 0.78, 95% [CI 0.63, 0.89]), suggesting that the probability of these correlation coefficients are more than 0 is 99.9% for all criteria. Regarding emotion-focused coping, we observed a negative association with criterion B (estimated correlation (p) = -0.39, 95% CI[-0.65, -0.09]), criterion C (estimated correlation (p) = -0.34, 95% CI[-0.62, -0.03]), and criterion D (estimated correlation (p) = -0.26, 95% CI[-0.56, 0.06]), suggesting that these estimated correlations are less than 0 by a probability of 99%, 97.8%, e 93.5%, respectively. However, part of the 95% HDI of these relationships fall in the ROPE. Finally, problem-focused coping had a negative association with criterion B (estimated correlation (p) = -0.28, 95% CI[-0.56, 0.06]), criterion C (estimated correlation (p) = -0.27, 95% CI[-0.57, 0.05]), and criterion D (estimated correlation (p) = -0.19, 95% CI[-0.51, 0.12]), suggesting that these correlations coefficients are less than 0 by a probability of 94.4%, 94.2%, and 87.3%, respectively. But again, part of the 95% HDI of all the estimated correlations (p) between PTSD criteria and problem-focused coping fall in the ROPE.

Since dysfunctional coping styles were the most reliable coping strategies being related to PTSD symptoms (the unique strategy in which the 95% HDI fell completely outside the ROPE), we further analyzed each coping subscale of the dysfunctional cluster to verify which one had the strongest correlation with PTSS. The Bayesian correlation test revealed a positive estimated correlation (p) of 0.64 between Self-Blame and PTSD symptoms (95% CI[0.42, 0.82]), suggesting that the correlation is more than 0 by a probability of 99.9%. The correlation (p) between Venting and PTSD symptoms was 0.44 (95% CI[0.15, 0.69]), suggesting that the correlation is more than 0 by a probability of 99.5%. Regarding Denial and PTSD symptoms, the estimated correlation (p) was 0.55 (95% CI[0.29, 0.77]), suggesting that the correlation is more than 0 by a probability of 99.9%. The correlations of Self-Distraction (estimated correlation (p) = 0.25, 95% CI[-0.09, 0.53]) and Behavioral Disengagement (estimated correlation (p) = 0.26, 95% CI[-0.05, 0.57]) with PTSD symptoms revealed positive estimated correlations with a probability of 92.7% and 93.8% that these correlations are more than 0, respectively. Finally, the Bayesian correlation test revealed an estimated correlation (p) of -0.003 between Substance Abuse and PTSD symptoms (95% CI[-0.35, 0.35]), suggesting a probability of 49.3% that this correlation is more than 0.

All the subscales estimated correlations (p) (Self-Distraction, Behavioral Disengagement, and Substance Abuse) had most of theirs 95% HDI in the ROPE, so more data is necessary to make a precise decision about their associations with PTSD symptoms. Self-Blame showed the largest effect size, suggesting that this strategy is the most prominent inside the dysfunctional coping cluster in its association with PTSS severity.

### Bayesian linear regression analysis

**Fig 2:**
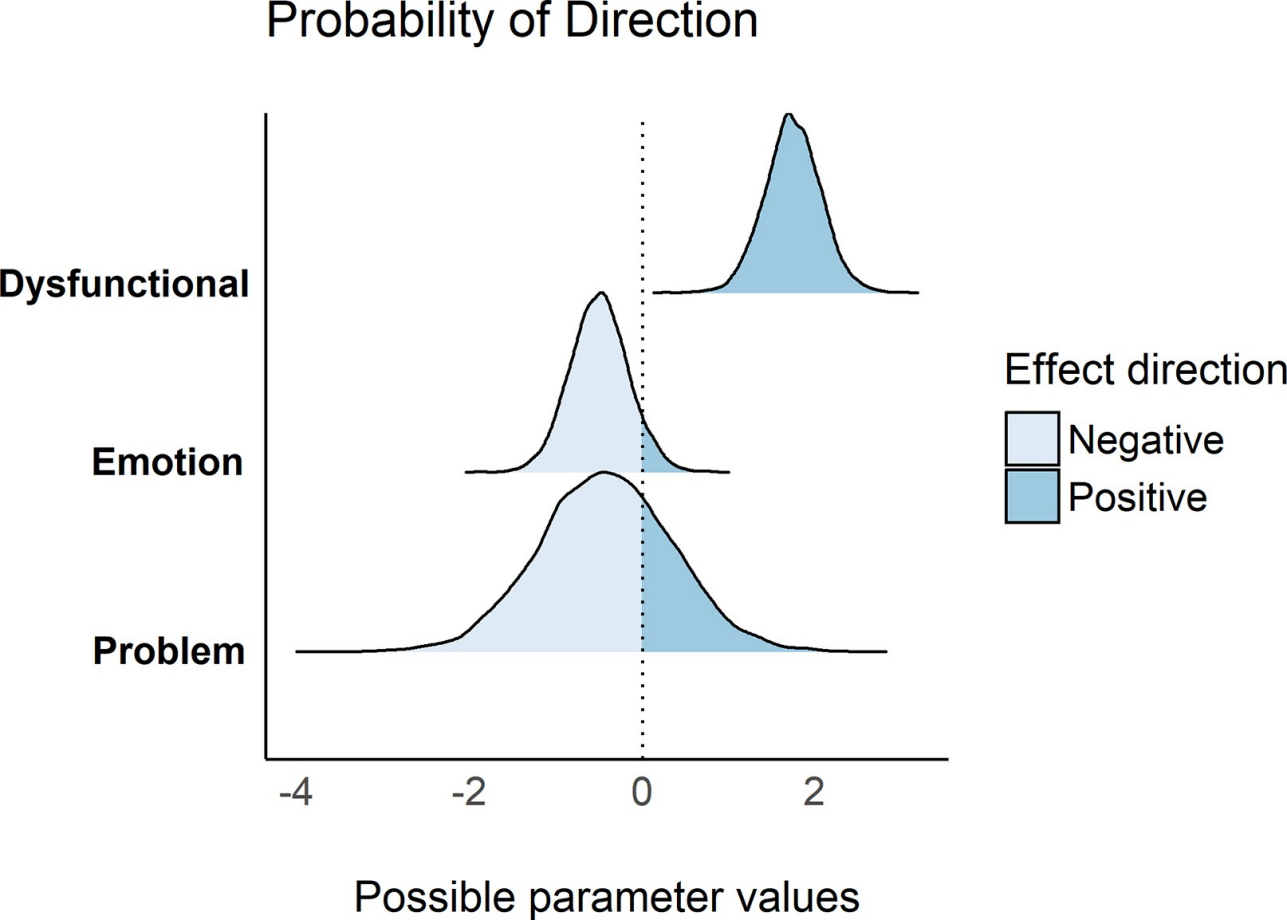
Posterior distributions of the beta coefficients of the coping strategies clusters included in the Bayesian regression model. This figure shows the probability of direction (positive or negative) of the posterior distribution of each coping strategy used in the Bayesian regression model.

We compared the Frequentist and Bayesian approach using the same model as the frequentist one. Thus, the model containing gender and the three coping strategies provided extreme (BF_10_ > 100) evidence in favor of this model (containing all variables) when compared to the null model (intercept only)–Table 5. As we can see, the point estimates of the slopes were very close to the frequentist regression. Only the dysfunctional coping style had its posterior distribution (95% CI[0.92, 2.22]) outside the region close to 0 –Fig 2, meaning that it is the main predictor of PTSS severity given our data.

**Table 5:** Bayesian linear regression analysis with PCL-C score as the dependent variable.

	$\hat{\beta }$ (posterior)	SD	95% CI		BF_10_
			Lower	Upper	
**Gender_male**	-1.44	1.87	-5.23	2.20	2005
**Gender_female**	1.44	1.87	-2.20	5.22	
**Dysfunctional coping**	1.58	0.33	0.93	2.22	
**Emotion-focused coping**	-0.43	0.32	-1.06	0.21	
**Problem-focused coping**	-0.42	0.73	-1.86	1.05	

Note. PCL-C = Posttraumatic Stress Disorder Checklist–Civilian Version. SD = Standard Deviation. CI = Credible Interval. BF_10_ = Bayes Factor in favor of the H_1_ (alternative hypothesis).

### Comparison between frequentist and Bayesian results

As we can see in Tables 6 and 7, we obtained similar r and beta values on the Bayesian approach when compared to the frequentist one. As we discussed in the following section, these similarities were expected since we used uninformative priors in the Bayesian analysis.

**Table 6:** Comparison between correlation coefficients of frequentist and Bayesian analysis.

Correlations	Frequentist		Bayesian	
	r	p-value	r (posterior)	95% CI
Dysfunctional coping x PCL-C	0.70	<0.001	0.71	0.51, 0.85
Emotion-focused coping x PCL-C	-0.36	0.03	-0.36	-0.63, -0.04
Problem-focused coping x PCL-C	-0.25	0.13	-0.27	-0.55, 0.04
Dysfunctional coping x Criterion B	0.56	<0.001	0.59	0.35, 0.79
Dysfunctional coping x Criterion C	0.60	<0.001	0.60	0.36, 0.79
Dysfunctional coping x Criterion D	0.79	<0.001	0.78	0.63, 0.89
Emotion-focused coping x Criterion B	-0.40	0.01	-0.39	-0.65, -0.09
Emotion-focused coping x Criterion C	-0.34	0.04	-0.34	-0.62, -0.03
Emotion-focused coping x Criterion D	-0.26	0.12	-0.26	-0.56, -0.06
Problem-focused coping x Criterion B	-0.25	0.14	-0.28	-0.56, 0.05
Problem-focused coping x Criterion C	-0.27	0.11	-0.27	-0.57, 0.05
Problem-focused coping x Criterion D	-0.19	0.27	-0.19	-0.51, 0.12

Note. r = correlation coefficient. CI = Credibility interval. PCL-C = Posttraumatic Stress Disorder Checklist–Civilian Version.

**Table 7:** Comparison between linear regression betas of frequentist and Bayesian analysis.

Independent variable	Frequentist		Bayesian	
	$\hat{\beta }$	p-value	$\hat{\beta }$ (posterior)	95% CI
Dysfunctional Coping	1.75	<0.001	1.58	0.93, 2.33
Emotion-focused coping	-0.49	0.15	-0.43	-1.06, 0.20
Problem-focused coping	-0.42	0.57	-0.42	-1.86, 1.05

Note. $\hat{\beta }$ = linear regression beta. CI = Credibility interval.

## Discussion

The present study aimed to investigate the relationship between habitual use of coping strategies and PTSS in a non-clinical sample of trauma-exposed college students whose trauma met the A criterion for PTSD diagnosis. The frequentist and Bayesian analysis showed that emotion-focused coping and dysfunctional coping were related to posttraumatic stress symptoms: emotion-focused coping was associated with less symptom severity, while dysfunctional coping were related with higher symptoms severity. Lastly, in both linear regression models, the cluster of dysfunctional coping was the only significant predictor of higher PTSS.

A variety of studies have explored the relationship between coping styles and PTSS. Rice et al. (2014) [11], for example, showed that in active duty members and military veterans, those with high PTSD scores used dysfunctional coping strategies (behavioral disengagement, venting and self-blame) more than those with low PTSD scores. In another study, Dworkin et al (2018) [30], reported that social support predicted reductions of PTSD symptoms in college women that were sexual assault survivors. However, to our knowledge, only Schnider et al. (2007) [16] investigated the relationship between coping strategies and PTSS in college students using the whole Brief COPE scale as an instrument. The authors used a non-clinical sample of college students who suffered a traumatic loss and reported that avoidant emotional coping was a strong predictor of PTSS severity. It is important to note that their cluster of avoidant emotional coping included all dysfunctional coping strategies of the Brief COPE except for venting. Here, we decided to use the nomenclature of “dysfunctional coping” instead of “avoidant coping” because it better describes the constructs contained in this cluster. Moreover, the authors found a positive association of emotion- and problem-focused coping styles with PTSS [16]. Similarly, in a study with nurses who had been exposed to Superstorm Sandy in 2012, Roberts et al. (2016) [7] observed that coping styles considered adaptive, such as active coping, instrumental support, and acceptance, were positively associated with PTSS severity. One of the explanations given is that in large-scale events, individuals tend to use more emotion and problem-focused coping styles as an attempt to eliminate the stressor or reduce stress [7].

In contrast to Schnider et al. (2007) and Roberts et al. (2016) [16,7], in our study we found that emotion-focused coping strategies were associated with reduced severity of PTSS. This finding is in line with the current literature finding that functional/adaptive coping styles are related to good health outcomes, such as low trait anxiety, high self-esteem, high heart rate variability, and low PTSS [9,24–25,56]. It is important to note that several studies measured coping styles associated with a specific traumatic event [7,9], whereas our study measured the usual coping styles of participants, which means that they completed the Brief COPE scale thinking about how they usually deal with stressors on a daily basis. Furthermore, one can speculate that individuals who use more adaptive coping styles—in this case, emotion-focused coping styles—tend to have a reduced psychological stress response. However, in both Frequentist and Bayesian regression models, emotion-focused strategies do not seem to not have a strong relation to PTSS severity. In fact, we can see that the posterior distribution of its beta coefficient is around the null value, so more data is necessary to draw better conclusions.

As expected, the cluster of dysfunctional coping was a strong predictor of PTSS severity. In fact, taking a closer look in the Bayesian analyses, it was the only coping cluster where the posterior distribution fell completely outside the null region, suggesting a higher probability that this coping style is the most strongly associated with PTSD symptoms. Moreover, as we can see the parameter’s posterior distribution (r), this correlation varies between a medium to a large effect size. Indeed, the association between PTSD and dysfunctional coping strategies is well known in the literature [9–11,15–16,19,27]. Badour and colleagues (2012) [27] observed a positive relationship between avoidant coping styles (e.g., denial, behavioral disengagement, and substance use) and PTSD severity among a sample of treatment-resistant veterans. The authors found that those who had higher scores in avoidant coping at the beginning of a residential rehabilitation program tended to have more severe PTSD at discharge. Moreover, having severe PTSD at discharge predicted greater avoidant coping styles at follow-up [27]. In line with these results, Rice et al. (2014) [11] found an association between dysfunctional coping styles and PTSD in a group of active duty service members and military veterans. Those who screened positive for PTSD tended to adopt more dysfunctional coping strategies (behavioral disengagement, self-blame, and venting) than those without PTSD. Moreover, these findings in clinical populations are consistent with our study showing that dysfunctional strategies are associated with higher PTSD symptoms in a non-clinical sample of trauma-exposed students.

The literature suggests that one explanation for this strong relationship is the conceptual and semantic overlap between dysfunctional coping strategies and criterion C (avoidance symptoms) of PTSD [16]. However, in the present study, the cluster of dysfunctional coping was associated with all three criteria of PTSD, suggesting that the association between dysfunctional coping and PTSS is not solely based on criterion C. These results are consistent with the findings of Schnider and colleagues (2007) [16]. In fact, we found a stronger correlation between dysfunctional coping style and criterion D (hyperarousal symptoms). Previous studies have shown that poor coping styles and disengagement coping styles are associated with lower vagal tone [56, 57]. One could hypothesize that these findings suggests an autonomic state of prominent sympathetic activation, which may help explain the strong relationship between dysfunctional coping and criterion D shown in the present study, since the criterion D involves symptoms of hyperarousal (e. g. insomnia and hypervigilance) that are associated with sympathetic activation. Aligned with these findings, studies have also shown that negative coping styles were related to higher levels of cortisol [58–59], suggesting increased activation of the hypothalamic-pituitary-adrenal (HPA) axis, which is also related to the arousal symptoms involved in criterion D [36]. Moreover, it is important to highlight that although self-distraction and denial coping styles are considered dysfunctional, there is some debate regarding whether these strategies can also be adaptive when the stressor can be avoided without future consequences, since these strategies would prevent exposure to the negative stimuli for a short time period [26,60]. Importantly, among all maladaptive coping strategies contained in the dysfunctional cluster, we observed that self-blame had the strongest correlation with posttraumatic stress symptoms, suggesting that stronger feelings of responsibility and auto criticism for events or situations on a daily basis can be the core of a maladaptive coping style. Some studies with female victims of rape and child abused are consistent with our results showing that self-blame is a strong construct involved in the severity of PTSS [61–62].

Despite the similar results between the frequentist and Bayesian approach (see Tables 6 and 7 in the results section), the Bayesian analysis have some advantages when compared to the frequentist method because it brings prior information to the model and it does not depend on p-values [63–66]. First, it was expected to find similar results between the two approaches in our study, since we used uninformative priors, which means that all possible values are equally likely to happen [63]. The selection of uninformative priors was made because this is the first study to use such approach to explore the association between PTSS and coping strategies. Moreover, future studies investigating the association between these two constructs could use our results as prior information to update their models. For example, if researchers are conducting a study exploring the relationship between dysfunctional coping and PTSS, they can include a prior information that this correlation is positive (e.g. r = 0 to 1). The second aspect in favor of the Bayesian approach is that the results obtained with the Bayesian method are more intuitive of its meaning [65]. As mentioned before, the Bayesian approach does not rely on p values (that are dependent on sample size) and uses sampling algorithms (MCMC), making it more robust to small samples and outliers [65–66]. Finally, the adoption of both, frequentist and Bayesian, methods of statistical inference provide us more information to draw better conclusions about the data [39].

Although our results contribute to the current literature, there were some limitations: (1) our study did not control for time and number of traumas; (2) since this is a cross-sectional study we cannot infer causality of coping styles on PTSS severity; (3) the use of PCL-C which uses the DSM-IV, a previous edition rather than the current one (PCL-5/DSM-V). Nonetheless, large differences between these scales are not expected; and (4) the final sample size was relatively small. However, the Bayesian approach used in the present study is less dependent on sample size when compared to classical tests that use p-values from null hypothesis tests [49,55].

In summary, the findings presented here show that, in a non-clinical sample of college students, those who use more dysfunctional coping styles have higher PTSS. Considering this, our results can be important to help guide the use and development of psychotherapeutic approaches around which coping styles to implement or avoid. This may potentially enhance the resilience of those exposed to traumatic events as well as diminish PTSD symptoms, which is central to survivors’ long-term mental health. The present findings add new data to the body of research regarding the mental health status of college students, a group that has been shown to have a relatively high incidence of psychiatric disorders such as anxiety and mood disorders [31–32,67].

## Supporting information

S1 File Study dataset. This file contains the Brief COPE and PCL-C scores for each participant used in the analyses.
(CSV)
